# Are we there yet? understanding interactions between respiratory viral infections, atopy, and the microbiome

**DOI:** 10.3389/falgy.2026.1952677

**Published:** 2026-09-18

**Authors:** Megan E. Hanna, Navya Kartha, Andriana LaMantia, Mitchell H. Grayson, Syed-Rehan A. Hussain

**Affiliations:** 1 Division of Allergy and Immunology, Department of Pediatrics, Nationwide Children’s Hospital - The Ohio State University College of Medicine, Columbus, OH, United States; 2 Center for Clinical and Translational Research, Abigail Wexner Research Institute at Nationwide Children’s Hospital, Columbus, OH, United States

**Keywords:** aeroallergen, atopy & microbiome, gut-lung aixs, rhinovirus (RV), RSV (respiratory syncytial virus)

## Abstract

Atopic disease has become an increasingly prevalent condition worldwide and a significant health concern. Similarly, due to concerns about morbidity associated with respiratory viral illness in early childhood, there has been increased exploration of the relationship between atopy and viral illnesses. Historically, studies have focused on early-life respiratory infections driving the development of atopy or post-viral airway disease, with respiratory syncytial virus (RSV) infection driving post-viral disease in those without atopy, while human rhinovirus (hRV) leading to asthma in those with pre-existing atopy. The impact of atopy on the antiviral immune response remains debated, with some studies suggesting it provides a protective role, while others suggest it impairs the immune response. Beyond the respiratory tract, the gut microbiome increasingly is recognized as a potential modulator of both local and distant immune responses, a relationship associated with lung function termed the gut-lung axis. As we continue to elucidate the biochemical mechanisms underlying these pathways in mouse models, it will be important to identify analogous human pathways. This review explores what is currently known about the relationship between atopy and respiratory viral infections focusing on knowledge about how viral infections drive atopy and, reciprocally, how atopy modulates respiratory viral induced disease. Moreover, we review published information on analogous human pathways. Utilizing this information will help us with better disease management and potential development of targeted therapeutics.

## Introduction

Atopic diseases are most prevalent in Western countries; however, their incidence continues to increase in low- and middle-income countries as urbanization and adoption of Western lifestyles expand (1). In 2024, allergic rhinitis was the most common atopic condition among Americans, affecting 25.2% of adults and 20.6% of children, followed by atopic dermatitis (7.7% of adults and 12.7% of children), asthma (8.7% of adults and 6.2% of children), and food allergies (6.7% of adults and 5.3% of children). Approximately 20% of the global population is affected by at least one atopic disease, and these conditions represent the most common chronic diseases of childhood (1–4). Beyond their high prevalence, atopic diseases impose a substantial economic burden, increase healthcare utilization, contribute to poorer health outcomes, and significantly diminish quality of life (5, 6). A better understanding of the mechanisms underlying their development is therefore critical for improving prevention and treatment strategies.

Respiratory viral infections are the leading cause of acute respiratory illness in infants and young children and have been associated with long-term respiratory morbidity, including recurrent wheezing, asthma, and recurrent respiratory infections (7). Observational studies have previously linked respiratory viral infections during infancy with an increased risk of allergic sensitization and subsequent atopic disease (8, 9). Consequently, respiratory viral infections have been proposed as a potential driver of allergic disease development (10). More recent investigations, however, suggest that the relationship between respiratory viral infection and atopy is more complex than previously appreciated. Emerging evidence suggests that immune pathways associated with atopy may protect against the development of post-viral airway disease, highlighting the need to better define the mechanisms underlying these interactions.

The increasing prevalence of atopic diseases is likely multifactorial, involving not only infectious triggers but also genetic susceptibility, the microbiome, and environmental exposures (5). Although much of the mechanistic evidence has been observed in animal models, a growing body of human studies has begun to validate many of these findings and reveal important similarities in the underlying immune pathways. This review summarizes the current evidence from both animal models and human studies on the development of atopic sensitization and allergic disease and highlights areas requiring further investigation and the implications for future therapeutic strategies.

## Impact of respiratory viral infections on atopy

Respiratory viral infections early in life have been associated with an increased risk of wheeze and even asthma. In early studies it was found that infants hospitalized with a severe RSV infection in the first 2–10 months of life were at a much-increased risk of developing asthma and even allergic sensitization. This same cohort was found to have increased atopic disease if they had severe RSV infection (11, 12). Being infected by RSV was insufficient to drive the atopic disease, as nearly all infants have been infected by RSV by 2 years of age. Other studies have demonstrated a seasonal relationship between recurrent wheeze, with increased recurrent wheeze in those infants born such that they were 2–6 months old during the RSV season (13, 14).

However, not all studies have shown a strong association between RSV and asthma, and indeed it seems that the stronger association is simply with the development of wheeze. In fact, studies have shown that this association is strongest shortly after the viral infection and appears to disassociate as the child ages (15–18).

Why RSV would have such an age restriction is curious. Most studies have proposed it is related to impaired Type I Interferon (IFN) production at these young ages, suggesting that increased type I IFN during the RSV infection might prevent the subsequent risk of wheeze (19). Other studies have demonstrated that the RSV induced risk for wheeze is only in those without pre-existing atopy, which could also explain the age predilection (20, 21). Unlike RSV, human Rhinovirus (hRV) has been demonstrated to drive the development of asthma and recurrent wheeze in those children who already have pre-existing atopy (and usually at an older age) (21–23) (Figure 1). The mechanisms by which these viruses (hRV and RSV) drive development of wheeze and/or atopic disease is still an active area of research, for which animal models are often used.

**Figure 1:**
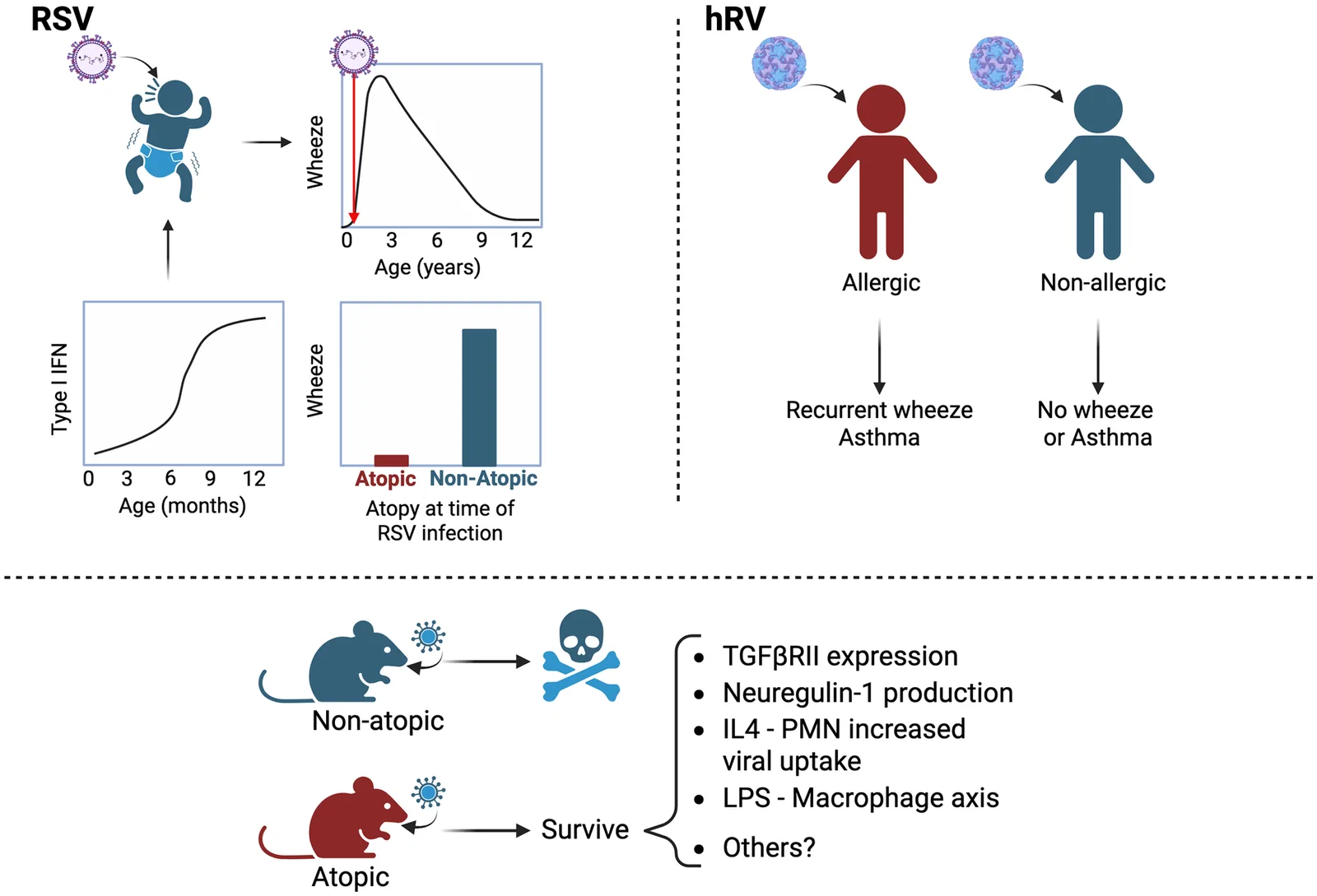
Schematic representation of the interaction between allergens and respiratory viruses. Top left panel: Infants before 6 months of age have impaired type I IFN responses and early life RSV infection of these infants leads to recurrent wheeze/asthma that tends to dissipate as the children age. The risk of wheeze in infants appears to be primarily only in those without pre-existing atopic disease. Top right panel: hRV drives recurrent wheeze and asthma in those children with pre-existing allergic disease. Bottom panel: In mouse models, pre-existing atopy has been shown to reduce post-viral airway disease and provide a survival advantage. The various pathways and factors that have been suggested to be important in this protection are listed. Created in BioRender. Grayson, M. (2026) https://BioRender.com/qxhcngg; License: CC-BY, Source: BioRender.

Animal models have suggested that infection with RSV drives type 2 innate lymphoid cells into the airways, which can lead to airway hyper-reactivity and mucous cell metaplasia (24, 25). However, RSV does not normally infect mice (it is not a natural rodent pathogen), which raises concerns over how applicable these findings are to humans. We and others have demonstrated that infection with murine parainfluenza virus-1 (Sendai virus, SeV, a natural rodent pathogen related to RSV) drives a well characterized pathway leading to post-viral airway disease (airway hyper-reactivity and mucous cell metaplasia) that likely lasts for the life of the animal (26–28).

This pathway starts with infection of the airway epithelium, which leads to recruitment of CD49d expressing neutrophils, who are maintained in the airway by LTD4 binding to CysLTR expressed on the neutrophils. These neutrophils then drive expression of FceRI, the high affinity receptor for IgE, on airway conventional dendritic cells (cDC). At the same time IgE is produced against SeV, and SeV is still present in the lungs. The crosslinking of anti-SeV IgE by SeV leads to production of CCL28 by the cDC. This recruits CCR10 expressing lymphocytes (Th2 and ILC2 cells), which produce IL-13 and drive the post-viral airway disease (29, 30). Exposure to a non-viral antigen in the middle of the antiviral immune response is sufficient to drive production of IgE against the non-viral antigen, suggesting the mice also develop atopy as a result of the infection (27). Over time NK cells interact with airway macrophages to drive an IL-13 dependent alternative activation of the macrophages. These macrophages then continue to produce IL-13 leading to the continued existence of the post-viral airway disease (31).

Components of these pathways have been demonstrated in humans—specifically the presence of CD49d expressing neutrophils with severe disease, production of IgE against respiratory viruses, increased cDC expression of FceRI, and production of CCL28 when FceRI is crosslinked on the cDC. While the mouse model is dependent upon production of antiviral IgE, it is not clear whether this applies to humans (28). Indeed, a human study found that treatment with anti-IgE reduced asthma exacerbations during respiratory viral seasons (32). The reduction in IgE was associated with an increased production of type I IFN from plasmacytoid dendritic cells (pDC), suggesting that atopy led to impaired antiviral immunity and that drove the asthma exacerbation (33–36). However, another study where subjects were treated with or without anti-IgE and then experimentally infected with hRV does not support this hypothesis. In this study, treatment with anti-IgE was associated with reduced asthma symptoms, but instead of having lower viral titers (presumably due to increased type I IFN) the subjects treated with anti-IgE had higher viral titers (37, 38). Clearly additional research is needed to better elucidate the relationship between respiratory viral infections and the development of atopy.

## Impact of atopy on respiratory viral infections

As indicated, atopy appears to have an ability to modulate the antiviral immune response in the lungs. Increased IgE will impair pDC production of type I IFN, although whether that has a significant effect on the antiviral immune response is less clear. There are some data to support the ability of atopy to be protective of severe respiratory infections in humans. In particular, during the 2009 H1N1 influenza pandemic having asthma was associated with reduced mortality and need for mechanical ventilation. Complicating this finding, though, was the fact that those with asthma received more systemic corticosteroids and were probably treated differently in the hospital than those without asthma (39, 40). During the COVID-19 pandemic several studies suggested that those with atopy had less severe disease than those without, and in fact some studies suggested that having atopy actually was protective from even getting COVID-19 (41).

Animal models have demonstrated a very significant effect of atopy on the lung antiviral immune response (Figure 1, bottom panel). Several studies have demonstrated that making mice atopic (primarily by using the house dust mite model) they become resistant to normally lethal infections with influenza or SeV (42–44). The mechanisms of this protection are less well defined. One study demonstrated that the protection required TGFbRII expression, while we initially found that neuregulin-1 is overproduced in atopic mice and treatment with neuregulin-1 in non-atopic mice protects them from a lethal viral infection (44, 45). However, our subsequent work suggests that the atopic effect on survival does not depend on (and is disparate from) neuregulin-1 (46). In fact, atopy might not be the critical factor in survival in the mouse models, as low dose lipopolysaccharide has been shown to provide a survival advantage in respiratory viral infection models, an effect that depended upon lung resident macrophages (47).

In addition to macrophages, neutrophils have been implicated in the atopic effect on the antiviral immune response. IL-4 treated neutrophils have increased phagocytosis, and we have shown that in atopic mice airway neutrophils contain increased genomic SeV RNA compared to neutrophils from non-atopic mice. Indeed, atopic mice have reduced SeV titers, and this effect can be reversed by depleting neutrophils or blocking IL-4 (48). This likely has significance for humans, as a study of preschool children found that the systemic neutrophil response to a viral infection was more effective in those with allergic disease (49).

## Microbiome, respiratory viral infections and atopy

Respiratory tract infections may alter the composition of the airway microbiome both during and after infection. Conversely, the existing airway microbiome may influence an individual's susceptibility to subsequent respiratory tract infections. During the first three years of life, hypopharyngeal colonization with *Streptococcus pneumoniae*, *Haemophilus influenzae*, or *Moraxella catarrhalis* has been associated with an increased risk of developing bronchitis and pneumonia at age four years. Early colonization with *Streptococcus* species has been associated with an increased risk of earlier onset of lower respiratory tract infections, whereas *Moraxella* colonization is associated with an earlier onset of upper respiratory tract infections (50) (Figure 2). The interactions between the microbiome and respiratory viruses leading to immunomodulation with an impact on the host response is an understudied area; however, emerging evidence suggests that this axis (microbiome-respiratory virus) may contribute to the pathogenesis of atopic diseases (51). Furthermore, commensal bacteria colonizing the respiratory tract have been reported to be altered in viral infection subsequently affecting the immune response and contributing to development of post-viral airway disease (52). Although it is worth noting that a recent prospective study of young children (mean age 8.63 yrs) with asthma found the nasal microbiome dominated by commensal bacteria, which provided greater protection against respiratory viral infection (53).

**Figure 2:**
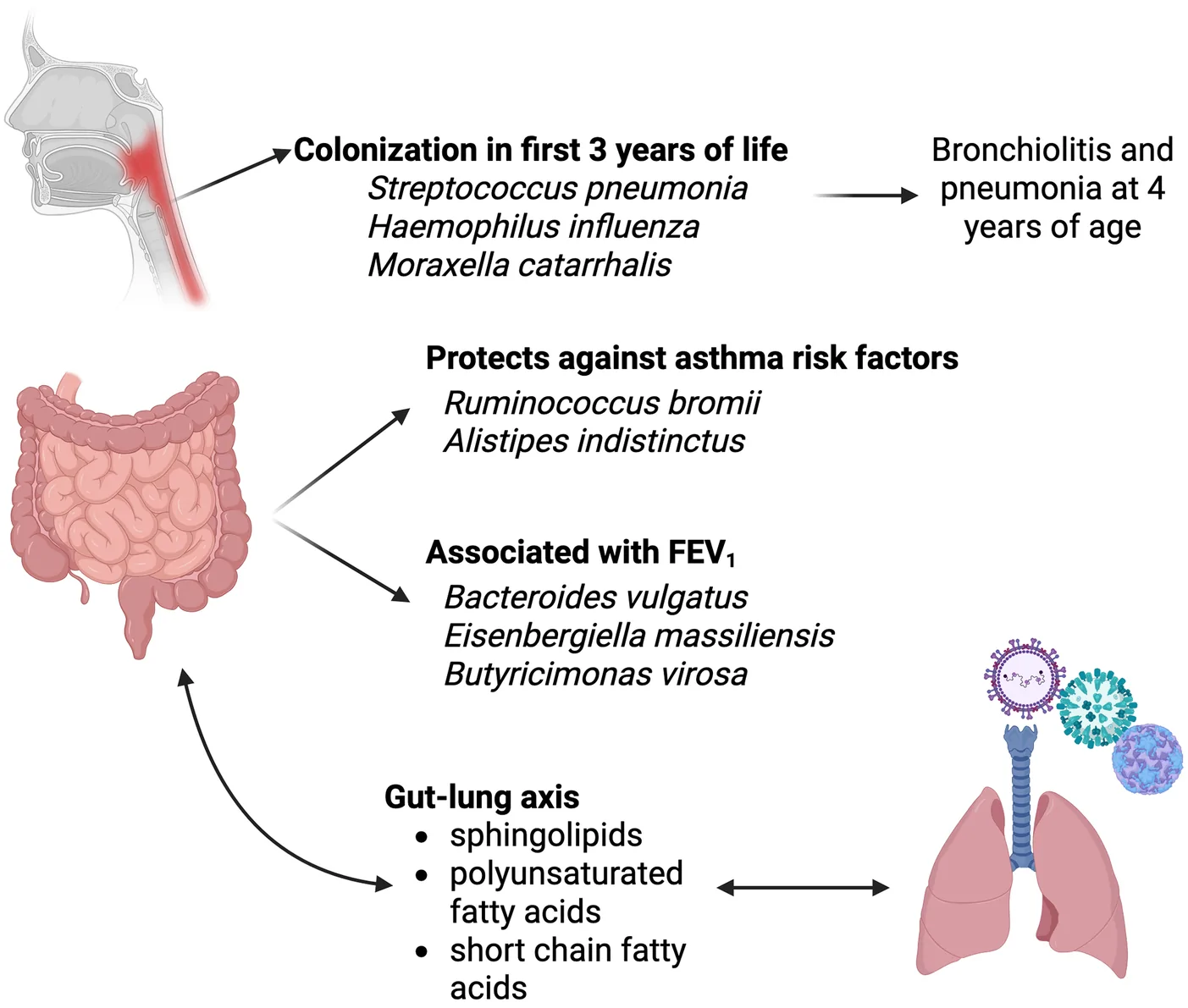
Microbiome affecting airway disease. Colonization by 3 years of age of the nasopharynx with *S. pneumoniae*, *H. influenzae*, and *M. catarrhalis* has been associated with the development of bronchiolitis and pneumonia by age 4 years. Specific microbiota in the gut has been associated with changes in airway function and asthma risk. Dysbiosis and reduced biodiversity of the gut microbiome can lead to increased susceptibility to respiratory viral infection due to modulation of metabolites and immune pathways through the gut-lung axis. Created in BioRender. Grayson, M. (2026) https://BioRender.com/v0oua63; License: CC-BY, Source: BioRender.

The influence of respiratory viruses, such as RSV, RV, IAV or SARS-CoV-2 on the nasopharyngeal and gut microbiome, or vice-versa, is unclear. Beyond the respiratory tract and environmental exposures, the gut microbiome has been increasingly recognized as a potential modulator of both local and distant immune responses, a relationship associated with lung function termed the gut-lung axis (54) (Figure 2). Furthermore, studies have evaluated how alterations in immune cells in response to viral infections can alter the gut microbiome. Earlier mouse studies discovered that CD8+ cell depletion increased food intake and prevented infection-induced anorexia which reversed gut microbiome changes normally seen in RSV. Furthermore, RSV infection also caused a shift in lipid metabolism due to changes in fecal gut metabolome along with an increase in sphingolipids, polyunsaturated fatty acids, and short chain fatty acids. All these factors may potentially be contributing to protective anti-inflammatory responses post viral exposure (55). Alternatively, when viruses interact with respiratory epithelium, they create a proinflammatory state which can shift towards Th2 inflammation leading to allergies and atopy (56). The proinflammatory state, as was seen in COVID-19, can lead to gut microbiome dysbiosis, which further exacerbates systemic inflammation (57). Recent publications have explored the alteration in commensal bacteria or respiratory microbiota when exposed to respiratory viruses leading to post viral lung injury possibly driving atopy. As with the case with influenza, specifically H1N1, there was a shift from *Actinobacter* to *Gammaproteobacteria,* which metabolize proinflammatory products leading to chronic lung diseases. Chronic inflammation driven by IL-13-mediated hypersecretion of mucus and airway hyperreactivity can be observed with influenza infection and post-RSV one year after infection. The virus-induced alteration in the respiratory tract microbiota may result in this shift towards a Th2 response and contribute to post-viral wheeze and asthma (52).

Studies have demonstrated that decreased microbiome biodiversity and dysbiosis are the largest contributors leading to decreased pathogenic microorganism clearance and immune system dysfunction, with increased dysbiosis contributing to more severe clinical atopic disease (54, 58). In patients affected by SARS-CoV-2, alterations in the gut microbiome occured due to cytokine storm leading to a leaky gut, which worsened both clinical presentation and long-term outcomes (59). Reduced gut microbiome diversity is also associated with decreased T regulatory cells and increased interferon gamma production, contributing to increased mortality with exposure to respiratory viral infections (60). Host gut microbiome analyses in children with a history of bronchiolitis demonstrated that specific bacterial species (i.e., *Bacteroides vulgatus, Eisenbergiella massiliensis*, and *Butyricimonas virosa*) were differentially associated with forced expiratory volume in one second (FEV1), while the presence of *Ruminococcus bromii* and *Alistipes indistinctus* were found to have a protective effect on children with risk factors for asthma (61).

While viral infections may alter the developing microbiome, emerging studies suggest that pre-existing microbial differences may also influence susceptibility to atopy. Landmark birth cohort studies have demonstrated that alterations in the early-life gut microbiome can precede development of childhood asthma, supporting a potential role for dysbiosis in shaping immune maturation and subsequent allergic risk (62). In addition, experimental evidence suggests that specific microbial taxa, including supplementation with Lactobacillus, may influence airway immune defense against both allergens and viral infection (63). Genetics play a role as well, as at least one study demonstrated that in infants without any microbial stimulation, only those with an inherited risk of asthma (mothers with asthma) developed asthma by 5 years of age (64). Together, these findings suggest that the microbiome may function also as important modifier of antiviral immunity and susceptibility to atopic disease. Metagenomic analyses of atopic patients have identified potential genetic influences on gut microbiome composition; however, these associations are regarded as a component of a larger complex, multifactorial, and dynamic ecosystem that is not yet comprehensively understood.

## Conclusion

As assessed in the review, there are multiple factors, including pre-existing atopy, the gut microbiome, and environmental factors, that contribute to the immune system's function. It is imperative to look beyond the initial hypothesis that early childhood viral infections drive development of atopy, but rather whether pre-existing atopy can serve as a protective evolutionary change to protect against significant morbidity and mortality with viral illness. As we continue to elucidate the biochemical mechanisms underlying these pathways in mouse models, it will be important to identify analogous human pathways and utilize this information to develop targeted therapeutic treatments when atopy becomes pathologic and viral illness becomes fatal. The aim of this review is to comprehensively discuss what has been researched in the field to suggest what gaps should be considered for exploration in the future. Although viral illnesses were previously thought to contribute to the development of atopy, emerging evidence suggests that immune pathways in previously sensitized individuals may confer protection against severe respiratory disease. Moreover, emerging evidence suggest that the gut-lung axis with gut microbiota communicating through immune pathways and metabolites such as short-chain fatty acids plays a pivotal role in modulating the axis between atopy and respiratory viral responses. Disruption of gut microbial balance has been linked to reduced pulmonary immunity and increased susceptibility to respiratory viral infections.

## References

[1] Thomsen SF. Epidemiology and natural history of atopic diseases. Eur Clin Respir J. (2015) 2. 10.3402/ecrj.v2.24642 PMC4629767 26557262

[2] Bottoms-McClain L Giri A Ng AE. Diagnosed Allergic Conditions in Adults: United States, 2024. NCHS Data Brief. 2026(545). 10.15620/cdc/174634 PMC12907992 41678829

[3] Ng AE Giri A Bottoms-McClain L. Diagnosed Allergic Conditions in Children Ages 0–17: United States, 2024. NCHS Data Brief. 2026(546). 10.15620/cdc/174635 PMC12914752 41678836

[4] CDC. Most Recent Asthma Data. Centers for Disease Control and Prevention (2026). Available online at: https://www.cdc.gov/asthma-data/about/most-recent-asthma-data.html (Accessed August 27, 2026)

[5] Martorano LM Grayson MH. Respiratory viral infections and atopic development: from possible mechanisms to advances in treatment. Eur J Immunol. (2018) 48(3):407–14. 10.1002/eji.201747052 29244204 PMC5844827

[6] Lopez-Tiro J Contreras-Contreras A Rodriguez-Arellano ME Costa-Urrutia P. Economic burden of severe asthma treatment: a real-life study. World Allergy Organ J. (2022) 15(7):100662. 10.1016/j.waojou.2022.100662 35833203 PMC9260620

[7] Teoh Z Conrey S McNeal M Burrell A Burke RM Mattison C. Burden of respiratory viruses in children less than 2 years old in a community-based longitudinal US birth cohort. Clin Infect Dis. (2023) 77(6):901–9. 10.1093/cid/ciad289 37157868 PMC10838707

[8] Peebles RS J. Viral infections, atopy, and asthma: is there a causal relationship? J Allergy Clin Immunol. (2004) 113(1 Suppl):S15–8. 10.1016/j.jaci.2003.10.033 14694345

[9] Martinez FD. Viruses and atopic sensitization in the first years of life. Am J Respir Crit Care Med. (2000) 162(3 Pt 2):S95–9. 10.1164/ajrccm.162.supplement_2.ras-8 10988160

[10] Sigurs N Bjarnason R Sigurbergsson F Kjellman B. Respiratory syncytial virus bronchiolitis in infancy is an important risk factor for asthma and allergy at age 7. Am J Respir Crit Care Med. (2000) 161(5):1501–7. 10.1164/ajrccm.161.5.9906076 10806145

[11] Sigurs N. A cohort of children hospitalised with acute RSV bronchiolitis: impact on later respiratory disease. Paediatr Respir Rev. (2002) 3(3):177–83. 10.1016/S1526-0542(02)00191-4 12376053

[12] Sigurs N Aljassim F Kjellman B Robinson PD Sigurbergsson F Bjarnason R. Asthma and allergy patterns over 18 years after severe RSV bronchiolitis in the first year of life. Thorax. (2010) 65(12):1045–52. 10.1136/thx.2009.121582 20581410

[13] Bont L Steijn M Van Aalderen WM Brus F Th Draaisma JM Van Diemen-Steenvoorde RA. Seasonality of long term wheezing following respiratory syncytial virus lower respiratory tract infection. Thorax. (2004) 59(6):512–6. 10.1136/thx.2003.013391 15170037 PMC1747053

[14] Buttarelli L Caselli E Gerevini S Leuratti P Gambadauro A Manti S. Predictive factors and clinical markers of recurrent wheezing and asthma after RSV infection. Viruses. (2025) 17(8):1073. 10.3390/v17081073 40872787 PMC12390669

[15] Kenmoe S Bowo-Ngandji A Kengne-Nde C Ebogo-Belobo JT Mbaga DS Mahamat G. Association between early viral LRTI and subsequent wheezing development, a meta-analysis and sensitivity analyses for studies comparable for confounding factors. PLoS One. (2021) 16(4):e0249831. 10.1371/journal.pone.0249831 33857215 PMC8049235

[16] Nguyen VT Roth K Engelhardt E Pearlstein A Davita TR Grayson MH. The usual suspects: respiratory syncytial virus and rhinovirus drive asthma development and exacerbation. Ann Allergy Asthma Immunol. (2025) 135(3):261–7. 10.1016/j.anai.2025.04.013 40287012 PMC12353342

[17] Rosas-Salazar C Chirkova T Gebretsadik T Chappell JD Peebles RS Dupont WD. Respiratory syncytial virus infection during infancy and asthma during childhood in the USA (INSPIRE): a population-based, prospective birth cohort study. Lancet. (2023) 401(10389):1669–80. 10.1016/S0140-6736(23)00811-5 37086744 PMC10367596

[18] van Wijhe M Johannesen CK Simonsen L Jørgensen IM Nair H Campbell H. A retrospective cohort study on infant respiratory tract infection hospitalizations and recurrent wheeze and asthma risk: impact of respiratory syncytial virus. J Infect Dis. (2022) 226(Suppl 1):S55–62. 10.1093/infdis/jiac141 35426942

[19] Hijano DR Vu LD Kauvar LM Tripp RA Polack FP Cormier SA. Role of type I interferon (IFN) in the respiratory syncytial virus (RSV) immune response and disease severity. Front Immunol. (2019) 10:566. 10.3389/fimmu.2019.00566 30972063 PMC6443902

[20] Simoes EA Carbonell-Estrany X Rieger CH Mitchell I Fredrick L Groothuis JR. The effect of respiratory syncytial virus on subsequent recurrent wheezing in atopic and nonatopic children. J Allergy Clin Immunol. (2010) 126(2):256–62. 10.1016/j.jaci.2010.05.026 20624638 PMC7126502

[21] Lukkarinen M Koistinen A Turunen R Lehtinen P Vuorinen T Jartti T. Rhinovirus-induced first wheezing episode predicts atopic but not nonatopic asthma at school age. J Allergy Clin Immunol. (2017) 140(4):988–95. 10.1016/j.jaci.2016.12.991 28347734 PMC7172285

[22] Jackson DJ Gangnon RE Evans MD Roberg KA Anderson EL Pappas TE. Wheezing rhinovirus illnesses in early life predict asthma development in high-risk children. Am J Respir Crit Care Med. (2008) 178(7):667–72. 10.1164/rccm.200802-309OC 18565953 PMC2556448

[23] Jartti T Kuusipalo H Vuorinen T Söderlund-Venermo M Allander T Waris M. Allergic sensitization is associated with rhinovirus-, but not other virus-, induced wheezing in children. Pediatr Allergy Immunol. (2010) 21(7):1008–14. 10.1111/j.1399-3038.2010.01059.x 20977499 PMC7168081

[24] Stier MT Goleniewska K Cephus JY Newcomb DC Sherrill TP Boyd KL. STAT1 represses cytokine-producing group 2 and group 3 innate lymphoid cells during viral infection. J Immunol. (2017) 199(2):510–9. 10.4049/jimmunol.1601984 28576981 PMC5505788

[25] Norlander AE Peebles RS Jr. Innate type 2 responses to respiratory syncytial virus infection. Viruses. (2020) 12(5):521. 10.3390/v12050521 32397226 PMC7290766

[26] Cheung DS Ehlenbach SJ Kitchens RT Riley DA Thomas LL Holtzman MJ. Cutting edge: cD49d+ neutrophils induce FcepsilonRI expression on lung dendritic cells in a mouse model of postviral asthma. J Immunol. (2010) 185(9):4983–7. 10.4049/jimmunol.1002456 20876348 PMC2959147

[27] Cheung DS Ehlenbach SJ Kitchens T Riley DA Grayson MH. Development of atopy by severe paramyxoviral infection in a mouse model. Ann Allergy Asthma Immunol. (2010) 105(6):437–43.e1. 10.1016/j.anai.2010.09.010 21130381 PMC3059126

[28] Cheung DS Sigua JA Simpson PM Yan K Hussain S-RA Santoro JL. Cysteinyl leukotriene receptor 1 expression identifies a subset of neutrophils during the antiviral response that contributes to postviral atopic airway disease. J Allergy Clin Immunol. (2018) 142(4):1206–17.e5. 10.1016/j.jaci.2017.11.026 29269317 PMC6005711

[29] Grayson MH Cheung D Rohlfing MM Kitchens R Spiegel DE Tucker J. Induction of high-affinity IgE receptor on lung dendritic cells during viral infection leads to mucous cell metaplasia. J Exp Med. (2007) 204(11):2759–69. 10.1084/jem.20070360 17954569 PMC2118483

[30] Thomas MA Buelow BJ Nevins AM Jones SE Peterson FC Gundry RL. Structure-function analysis of CCL28 in the development of post-viral asthma. J Biol Chem. (2015) 290(7):4528–36. 10.1074/jbc.M114.627786 25556652 PMC4326855

[31] Byers DE Holtzman MJ. Alternatively activated macrophages and airway disease. Chest. (2011) 140(3):768–74. 10.1378/chest.10-2132 21896520 PMC3168852

[32] Busse WW Morgan WJ Gergen PJ Mitchell HE Gern JE Liu AH. Randomized trial of omalizumab (anti-IgE) for asthma in inner-city children. N Engl J Med. (2011) 364(11):1005–15. 10.1056/NEJMoa1009705 21410369 PMC3093964

[33] Tversky JR Le TV Bieneman AP Chichester KL Hamilton RG Schroeder JT. Human blood dendritic cells from allergic subjects have impaired capacity to produce interferon-alpha via toll-like receptor 9. Clin Exp Allergy. (2008) 38(5):781–8. 10.1111/j.1365-2222.2008.02954.x 18318750 PMC2707903

[34] Gill MA Liu AH Calatroni A Krouse RZ Shao B Schiltz A. Enhanced plasmacytoid dendritic cell antiviral responses after omalizumab. J Allergy Clin Immunol. (2018) 141(5):1735–43.e9. 10.1016/j.jaci.2017.07.035 28870461 PMC6013066

[35] Becker Y. Respiratory syncytial virus (RSV) evades the human adaptive immune system by skewing the Th1/Th2 cytokine balance toward increased levels of Th2 cytokines and IgE, markers of allergy–a review. Virus Genes. (2006) 33(2):235–52. 10.1007/s11262-006-0064-x 16972040

[36] Coenen I Jones AC White AA Takashima M Lee WR Wong MD. Reduced type-I interferon by plasmacytoid dendritic cells and asthma in school-aged children. Allergy. (2026) 81(1):109–20. 10.1111/all.70005 40799176 PMC12773650

[37] Heymann PW Platts-Mills TAE Woodfolk JA Borish L Murphy DD Carper HT. Understanding the asthmatic response to an experimental rhinovirus infection: exploring the effects of blocking IgE. J Allergy Clin Immunol. (2020) 146(3):545–54. 10.1016/j.jaci.2020.01.035 32018030 PMC7733370

[38] Esquivel A Busse WW Calatroni A Togias AG Grindle KG Bochkov YA. Effects of omalizumab on rhinovirus infections, illnesses, and exacerbations of asthma. Am J Respir Crit Care Med. (2017) 196(8):985–92. 10.1164/rccm.201701-0120OC 28608756 PMC5649984

[39] Myles PR Semple MG Lim WS Openshaw PJM Gadd EM Read RC. Predictors of clinical outcome in a national hospitalised cohort across both waves of the influenza A/H1N1 pandemic 2009–2010 in the UK. Thorax. (2012) 67(8):709–17. 10.1136/thoraxjnl-2011-200266 22407890 PMC3402749

[40] Veerapandian R Snyder JD Samarasinghe AE. Influenza in asthmatics: for better or for worse? Front Immunol. (2018) 9:1843. 10.3389/fimmu.2018.01843 30147697 PMC6095982

[41] Seibold MA Moore CM Everman JL Williams BJM Nolin JD Fairbanks-Mahnke A. Risk factors for SARS-CoV-2 infection and transmission in households with children with asthma and allergy: a prospective surveillance study. J Allergy Clin Immunol. (2022) 150(2):302–11. 10.1016/j.jaci.2022.05.014 35660376 PMC9155183

[42] Ishikawa H Sasaki H Fukui T Fujita K Kutsukake E Matsumoto T. Mice with asthma are more resistant to influenza virus infection and NK cells activated by the induction of asthma have potentially protective effects. J Clin Immunol. (2012) 32(2):256–67. 10.1007/s10875-011-9619-2 22134539 PMC3305878

[43] An S Jeon YJ Jo A Lim HJ Han YE Cho SW. Initial influenza virus replication can be limited in allergic asthma through rapid induction of type III interferons in respiratory epithelium. Front Immunol. (2018) 9:986. 10.3389/fimmu.2018.00986 29867963 PMC5966536

[44] Furuya Y Furuya AK Roberts S Sanfilippo AM Salmon SL Metzger DW. Prevention of influenza virus-induced immunopathology by TGF-beta produced during allergic asthma. PLoS Pathog. (2015) 11(9):e1005180. 10.1371/journal.ppat.1005180 26407325 PMC4583434

[45] Hussain S-RA Rohlfing M Santoro J Chen P Muralidharan K Bochter MS. Neuregulin-1 prevents death from a normally lethal respiratory viral infection. PLoS Pathog. (2025) 21(4):e1013124. 10.1371/journal.ppat.1013124 40267147 PMC12052188

[46] Li Y Ghobashi A Hussain S-RA Rohlfing M Santoro J Bochter MS. Single-cell RNA sequencing of hematopoietic and non-hematopoietic cells defines a distinct signature in atopic and NRG1 mouse lung. Sci Rep. (2026). 10.1038/s41598-026-61413-w PMC13649604 42432033

[47] Resiliac J Rohlfing M Santoro J Hussain SA Grayson MH. Low-dose lipopolysaccharide protects from lethal paramyxovirus infection in a macrophage- and TLR4-dependent process. J Immunol. (2023) 210(3):348–55. 10.4049/jimmunol.2200604 36480273 PMC9851983

[48] Hussain S-RA Rohlfing M Resiliac J Santoro J Peeples ME Garcin D. Atopic neutrophils prevent postviral airway disease. J Immunol. (2021) 207(10):2589–97. 10.4049/jimmunol.2100766 34625522 PMC8578464

[49] Fitzpatrick AM Mohammad AF Huang M Stephenson ST Patrignani J Kamaleswaran R. Functional immunophenotyping of blood neutrophils identifies novel endotypes of viral response in preschool children with recurrent wheezing. J Allergy Clin Immunol. (2023) 152(6):1433–43. 10.1016/j.jaci.2023.08.010 37604313 PMC10841272

[50] Huang YJ Lynch SV. The emerging relationship between the airway microbiota and chronic respiratory disease: clinical implications. Expert Rev Respir Med. (2011) 5(6):809–21. 10.1586/ers.11.76 22082166 PMC3359942

[51] Mageiros L Megremis S Papadopoulos NG. The virome in allergy and asthma: a nascent, ineffable player. J Allergy Clin Immunol. (2023) 152(6):1347–51. 10.1016/j.jaci.2023.09.022 37778473

[52] Stadler SV Von Garnier C Ubags ND. Post-viral lung diseases: the microbiota as a key player. ERJ Open Res. (2025) 11(2):00560-2024. 10.1183/23120541.00560-2024 40196711 PMC11973713

[53] Kelly MS Huang C-Y Kim M Haghnazari D Baig A Sun Y. Nasal microbiome and phageome profiles are associated with prospective respiratory viral infection risk in school-age children. J Allergy Clin Immunol. (2026) 158(2):408–20. 10.1016/j.jaci.2026.01.017 41633490 PMC12900554

[54] Hufnagl K Pali-Scholl I Roth-Walter F Jensen-Jarolim E. Dysbiosis of the gut and lung microbiome has a role in asthma. Semin Immunopathol. (2020) 42(1):75–93. 10.1007/s00281-019-00775-y 32072252 PMC7066092

[55] Groves HT Higham SL Moffatt MF Cox MJ Tregoning JS. Respiratory viral infection alters the gut microbiota by inducing inappetence. mBio. (2020) 11(1):e03236-19. 10.1128/mBio.03236-19 32071269 PMC7029140

[56] Kloepfer KM Kennedy JL. Childhood respiratory viral infections and the microbiome. J Allergy Clin Immunol. (2023) 152(4):827–34. 10.1016/j.jaci.2023.08.008 37607643 PMC10592030

[57] Martin Gimenez VM Modrego J Gomez-Garre D Manucha W De Las Heras N. Gut microbiota dysbiosis in COVID-19: modulation and approaches for prevention and therapy. Int J Mol Sci. (2023) 24(15):12249. 10.3390/ijms241512249 37569625 PMC10419057

[58] Pantazi AC Mihai CM Balasa AL Chisnoiu T Lupu A Frecus CE. Relationship between gut microbiota and allergies in children: a literature review. Nutrients. (2023) 15(11):2529. 10.3390/nu15112529 37299492 PMC10255222

[59] Hendricks SA Vijayam B. The interplay between oral, nasal, lungs and gut microbiome ecology in coronavirus disease 2019 (COVID-19) infection. Semarak Int J Public Health Primary Care. (2025) 1(1):16–36. 10.37934/sijphpc.1.1.1636b

[60] Grayson MH Camarda LE Hussain S-RA Zemple SJ Hayward M Lam V. Intestinal microbiota disruption reduces regulatory T cells and increases respiratory viral infection mortality through increased IFNgamma production. Front Immunol. (2018) 9:1587. 10.3389/fimmu.2018.01587 30042764 PMC6048222

[61] Miyachi H Shibata R Javornik Cregeen SJ Surathu A Sijaric M Espinola JA. Interactions between host genetics and gut microbiome influence susceptibility to childhood asthma and lung function. J Allergy Clin Immunol. (2026) 157(4):868–78. 10.1016/j.jaci.2025.12.1005 41485494 PMC13135221

[62] Arrieta M-C Stiemsma LT Dimitriu PA Thorson L Russell S Yurist-Doutsch S. Early infancy microbial and metabolic alterations affect risk of childhood asthma. Sci Transl Med. (2015) 7(307):307ra152. 10.1126/scitranslmed.aab2271 26424567

[63] Fujimura KE Demoor T Rauch M Faruqi AA Jang S Johnson CC. House dust exposure mediates gut microbiome lactobacillus enrichment and airway immune defense against allergens and virus infection. Proc Natl Acad Sci U S A. (2014) 111(2):805–10. 10.1073/pnas.1310750111 24344318 PMC3896155

[64] Stokholm J Blaser MJ Thorsen J Rasmussen MA Waage J Vinding RK. Maturation of the gut microbiome and risk of asthma in childhood. Nat Commun. (2018) 9(1):141. 10.1038/s41467-017-02573-2 29321519 PMC5762761

